# Depletion of multidrug‐resistant uropathogenic *Escherichia coli* BC1 by ebselen and silver ion

**DOI:** 10.1111/jcmm.15920

**Published:** 2020-09-25

**Authors:** Peng Wang, Jun Wang, Zonglan Xie, Jingxuan Zhou, Qianqian Lu, Ying Zhao, Chuanjiang Dong, Lili Zou

**Affiliations:** ^1^ The First College of Clinical Medical Science China Three Gorges University Yichang, Hubei China; ^2^ The Institute of Infection and Inflammation Medical College China Three Gorges University Yichang, Hubei China; ^3^ Central Laboratory The People’s Hospital of China Three Gorges University Yichang, Hubei China; ^4^ Key Laboratory of Luminescent and Real‐Time Analytical Chemistry (Southwest University) Ministry of Education College of Pharmaceutical Sciences Southwest University Chongqing China

**Keywords:** ebselen, glutathione, redox homeostasis, silver ion, thioredoxin reductase, uropathogenic *Escherichia coli*

## Abstract

Ebselen, an organo‐selenium compound with well‐characterized toxicology and pharmacology, recently exhibited potent antibacterial activity against glutathione (GSH)‐negative bacteria by disrupting redox homeostasis. In this paper, we show that ebselen and silver ion in combination exert strong bactericidal activity against multidrug‐resistant (MDR) uropathogenic *Escherichia coli* (UPEC) BC1, a model MDR GSH‐positive bacterium. The mechanisms were found to involve consumption of total intracellular GSH and inhibition of thioredoxin reductase activity, which was highly related to reactive oxygen species up‐regulation. Furthermore, the therapeutic efficacy of ebselen and silver ion against UPEC‐induced cystitis was assessed in a mouse model. Treatment with ebselen and silver ion significantly reduced bacterial loads, down‐regulated the expression levels of tumour necrosis factor‐α (TNF‐α) and interferon‐γ (IFN‐γ) on‐site and decreased white/red blood cell counts in mild cystitis model mice, which demonstrated the anti‐inflammatory property of these agents. In addition, ebselen and silver ion also exhibited significantly high protective ability (100%) against acute cystitis infections. These results together may lay the foundation for further analysis and development of ebselen and silver ion as antibacterial agents for treatment of MDR UPEC infections.

## 1 INTRODUCTION

Bladder infection caused by uropathogenic *Escherichia coli* (UPEC) affects ~150 million people annually,^1^, ^2^ which accounts for up to 75% of all cases and 95% of community‐acquired cases.^3^, ^4^ UPEC‐induced urinary tract infection (UTI) may lead to potentially fatal bloodstream infection.^5^, ^6^ Despite effective antibiotic therapy, 30%‐50% of patients experience recurrent UTI (rUTI), and women are at significantly greater risk.^5^, ^7^ This places a heavy financial burden on the healthcare system, costing up to 6 billion USD for treatment and management each year.^6^ The growing prevalence of UPEC resistant to last‐line antibiotic treatments, and more recently carbapenems and colistin, makes cystitis a prime example of the antibiotic resistance crisis, emphasizing the need for new approaches to treat and prevent the related infections.^8^, ^9^

There are two major cellular thiol‐dependent redox systems, the thioredoxin (Trx) and glutathione (GSH) systems, which maintain cellular redox homeostasis and protect cells from oxidative stress.^10^, ^11^ The Trx system is composed of Trx, thioredoxin reductase (TrxR) and NADPH, while glutaredoxin (Grx), glutathione reductase (GR), GSH and NADPH constitute the GSH/Grx system.^12^, ^13^ Both systems are important in DNA synthesis and repair, cell proliferation and antioxidant defences.^14^, ^15^ The Trx system is widespread in all living organisms, and the GSH/Grx system is absent in the majority of Gram‐positive bacteria (GSH‐negative bacteria).^14^, ^16^ Thus, the maintenance of intracellular redox balance in GSH‐negative bacteria is principally dependent on the Trx system. It is worth noting that in the pathological process of UTI, UPEC infection reduces the levels of antioxidant enzymes including GR and glutathione peroxidase (GPx) in urinary tissue. In the late stage of UPEC infection, the levels of GSH decrease, while lipid peroxidation increases significantly in the urinary tissue.^17^ This decline in antioxidant enzymes provides a more suitable microenvironment for elevating the ROS level in the urinary system, leading to cell and tissue damage and increasing the severity of bacterial infection.

As an organo‐selenium compound, 2‐phenyl‐1,2 benzisoselenazol‐3(2H)‐one (ebselen) is known to be a classic GPx mimic and a substrate of mammalian TrxR.^16^ It is also a clinical trial drug with well‐characterized toxicology and pharmacology for stroke, neurodegenerative disease, bipolar disorder, COVID‐19, tobramycin‐induced ototoxicity, chemotherapy‐induced hearing loss.^18^, ^19^ Previous researches have shown that ebselen can protect against oxidative injuries in various tissues,^20^, ^21^, ^22^ and recent studies have demonstrated that ebselen possesses antibacterial activity against GSH‐negative bacteria.^16^, ^18^, ^23^, ^24^ Our group further confirmed that ebselen could work synergistically with silver ion to kill types of GSH‐positive bacteria,^18^ including multidrug‐resistant (MDR) *E coli*, and the effective concentration of silver ion was significantly reduced. Despite the gradual increase of reports concerning the antibacterial activity of ebselen and silver ion, few studies have focused on its involvement in bacteria‐caused host inflammation. In this paper, the bactericidal activity of ebselen and silver ion against the MDR UPEC BC1 strain has been detected in vitro by visible spectrophotometry, DTNB, flow cytometry, qPCR and Western blot assays. Furthermore, the mild and acute mouse cystitis models were constructed and the therapeutic efficacy, the regulation of UPEC‐induced inflammatory properties and the potential clinical applications of ebselen and silver ion against MDR UPEC were also assessed.

## 2 MATERIALS AND METHODS

### 2.1 Mouse and bacterial strains

BALB/c mice (male, 18‐22 g) were purchased from China Three Gorges University, and approval of the Medical Animal Care & Welfare Committee of China Three Gorges University was obtained prior to using the mice for study. Every five mice were kept in one cage individually with a constant dark‐light cycle in a conventional SPF animal house and were given free access to food and water.

*E coli* BC1 strain was isolated from patients with UTI at First Clinical Hospital of Yichang (the First College of Clinical Medical Science, China Three Gorges University), with an approval and written informed consent for research from the Ethics Committee of the First Affiliated Hospital of China Three Gorges University and informed consent of the patient. The strain was identified as an MDR UPEC^25^ (Table 1) by biochemical identification and MALDI‐TOF‐MS analysis, which is only sensitive to amikacin and aztreonam. All the experiments were performed in a BSL2‐plus laboratory.

**TABLE 1:** Biochemistry identification of clinical isolated UPEC BC1

2 AMY	−	16 BGAR	−	28 AlaA	−	44 NAG	+	−	−
4 PIPLC	−	17 AMAN	−	29 TyrA	−	45 dMAL	+	58 O129R	+
5 DXY	−	19 dSOR	−	30 dSOR	+	46 BACI	+	59 SAL	−
8 ADH1	+	20 LeuA	−	31 URE	+	47 NOVO	−	60 SAC	+
9 BGAL	+	23 PROA	−	32 POLYB	−	50 NC6.5	+	62 dTRE	+
11 AGLU	+	24 BGURr	−	37 dGAL	+	52 dMAN	+	63 ADH2s	+
13 APPA	−	25 AGAL	−	38 dRIB	+	53 dMNE	+	64 OPTO	+
14 CDEX	−	26 PyrA	+	39 ILATK	+	54 MBdG	+		
15 AspA	−	27 BGUR	−	42 LAC	−	56 PUL	−		

Abbreviations: ADH1, Arginine double hydrolase 1; ADH2s, Arginine double hydrolase 2; AGAL, α‐galactosidase; AGLU, α‐glucosidase; AlaA, Alanine aromatase; AMAN, α‐Mannosidase; AMY, Amygdalin; APPA, Alanine‐phenylalanine‐proline aromaminase; AspA, L‐aspartate arylamine; BACI, Bacillus peptide tolerance; BGAL, β‐D‐galactosidase; BGAR, β‐galactopyranosidase; BGUR, β‐D‐glucuronidase; BGURr, β‐glucuronidase; CDEX, Cyclodextrin; dGAL, D‐galactose; dMAN, D‐mannitol; dMNE, D‐mannose; dRAF, D‐raffinose; dRIB, D‐ribose; dSOR, D‐sorbitol; dTRE, D‐trehalose; dXYL, D‐xylose; LAC, Lactose; LeuA, Leucine aromaminase; lLATk, Lactate produces alkali; MBdG, Methyl‐B‐D‐glucopyranoside; NAG, N‐acetyl‐D‐glucosamine dMAL D‐Maltose; NC6.5,6.5% NaCl growth; NOVO, Novomycin tolerance; O129R, O/129 tolerant; OPTO, Optoxin tolerance; PHOS, Phosphatase; PIPLC, Phosphatidylphosphatidase C; POLYB, Polycolistin B tolerance; ProA, Proline aromaminase; PUL, Pullulan; PyrA, Pyroglutaminase; SAC, Saccharose; SAL, Salicin; TyrA, Tyrosine aromaminase; URE, Urease.

### 2.2 Reagents

Bacterial cells were cultured in Luria Bertani (LB) medium (EMD Millipore). 2‐phenyl‐1, 2‐benzisoselenazol‐3(2H)‐one (ebselen) (Daiichi), protease inhibitor cocktails (Roche), *E coli* DHB4 Trx protein and anti‐*E coli* Trx1 polyclonal antiserum were obtained from IMCO Corp. (Stockholm, Sweden; http://www.imcocorp.se). Rabbit anti‐sheep IgG‐HRP, goat anti‐mouse H&L, anti‐TNF‐α, anti‐IFN‐γ and anti‐DnaK antibodies were from Santa Cruz. IgG2a mouse monoclonal antibody was from VIROGEN, Lowry protein assay was from Bio‐Rad DC™, and all the other reagents were from Sigma‐Aldrich.

### 2.3 The inhibition of UPEC BC1 growth by ebselen and silver ion

The inhibition of UPEC BC1 growth by ebselen and silver ion was measured by visible spectrophotometer at 600 nm. UPEC BC1 cells were grown (37°C, 220 rpm) 8 hours and diluted 100 times that to be treated with serial concentrations of ebselen and silver ion for 16, 20 and 24 hours at 37°C in a 96‐well plate, and the absorbance values at 600 nm were measured. ceftazidime was used as negative control, and amikacin was used as positive control.

### 2.4 The disruption of UPEC BC1 cell membrane by ebselen and silver ion

The inhibition efficiency of ebselen and silver ion against UPEC BC1 was detected by Nuclear staining reagent propidium iodide (PI). BC1 cells were grown (37°C, 220 rpm) until an OD_600 nm_ of 0.4, which were treated with 80 μmol/L ebselen and 5 μmol/L silver ion (1.09 mmol/L amikacin was used as positive control; 1.17 mmol/L ceftazidime was used as negative control) for 20 minutes at 37°C. Cells were stained with 5 μg/mL PI for 30 min at 37°C , and the fluorescence was measured by flow cytometry (BECKMAN COULTER, AW15093).

### 2.5 Effect of ebselen and silver ion on UPEC BC1 bacterial morphology

UPEC BC1 was grown until an OD_600 nm_ of 0.4 and separately treated for 20 minutes with 80 μmol/L ebselen and 5 μmol/L silver ion, 80 μmol/L ebselen, 5 μmol/L silver ion, 1.09 mmol/L amikacin, 1.17 mmol/L ceftazidime and PBS. Cells were obtained by centrifugation (4°C, 13 000 rpm, 15 minutes) and fixed with 2.5% glutaraldehyde. The morphology and structure of *E coli* cells were observed by transmission electron microscopy (Hitachi H‐7500).

### 2.6 The inhibition of UPEC BC1 TRXR activity and depletion of GSH level by ebselen and silver ion

UPEC BC1 cells were cultured until an OD_600 nm_ of 0.4 and incubated with 80 μmol/L ebselen and 5 μmol/L silver ion for 20 minutes. BC1 cells were obtained by centrifugation (4°C, 5000 rpm, 5 minutes), and a protein inhibitor cocktail ( in 50 mmol/L Tris‐EDTA buffer, pH 7.4) was added to decrease the protease activity. The cells were disrupted with sonication (240 W, 5 minutes), and the supernatants were obtained by centrifugation (4°C, 12 000 rpm, 10 minutes). The TrxR activity and GSH amount assays were performed in a 96‐well plate. The activity of the untreated group was considered to be 100%. The protocols were performed as previously described.^18^, ^26^, ^27^

### 2.7 Trx1 mRNA expression in UPEC BC1 treated with ebselen and silver ion

UPEC BC1 cells were cultured until an OD_600 nm_ of 0.4 and incubated with 80 μmol/L ebselen and 5 μmol/L silver ion for 20 minutes. The total RNA was extracted and was reverse transcribed to cDNA. Real‐time fluorescence PCR (qPCR) analysis was performed with a PCR Bio‐Rad CFX96 Touch™ system. The primers for *trxa* and *rrs* used in qPCR are listed in Table 2, and the cycling protocol included an initial denaturation at 95°C for 30 seconds followed by 40 cycles of 95°C for 5 seconds, 60°C for 30 seconds and 79°C for 10 seconds. Finally, a melting curve was constructed to ensure that there was no contamination. qPCR of the genes of interest, *trxa*, and a normalizer gene, *rrs*, were performed in triplicate for each sample and included a no template control to rule out contamination and primer‐dimers' formation. The expression fold change of *trxa* was calculated based on the comparison with the normalized *rrs*.

**TABLE 2:** Antimicrobial susceptibility test of UPEC BC1

Antibiotics	Diameter (mm)	Cut‐off	Sensitivity/resistance
Gentamicin	23	12‐15	(S)
Levofloxacin	28	15‐19	(S)
Ciprofloxacin	24	15‐21	(S)
Selectin	30	10‐16	(S)
Tetracycline	14	14‐19	(R)
Penicillin	10	28‐29	(R)
Oxacillin	6	10‐13	(R)
Vancomycin	0.38	2‐16	(S)
Erythromycin	6	13‐23	(R)
Clindamycin	6	14‐21	(R)
Rifampicin	31	16‐20	(S)
Linezolid	31	20‐21	(S)
Chloromycetin	26	12‐18	(S)

### 2.8 Trx1 protein expression level in UPEC BC1 treated with ebselen and silver ion

UPEC BC1 cells were cultured until an OD_600 nm_ of 0.4, which were incubated with 80 μmol/L ebselen and 5 μmol/L silver ion for 20 minutes. Trx1 protein expression of BC1 cells treated with 80 μmol/L ebselen and 5 μmol/L silver ion was detected by Western blotting. After lysis by sonication, the cell lysates were obtained by centrifugation at 13 000 rpm for 20 minutes. Western blotting assay was performed with anti‐*E coli* Trx1 polyclonal antiserum (anti‐Dank antibody was used as reference). The normalized Trx1 levels with respect to the reference DnaK have been presented.

### 2.9 Protein *S*‐glutathionylation in UPEC BC1 treated with ebselen and silver ion

Total protein *S*‐glutathionylation (*S*‐PSSG) of 80 μmol/L ebselen and 5 μmol/L silver ion‐treated *E coli* cells were detected by Western blotting. Cells were cultured, washed and resuspended in lysis buffer (as described above) containing 30 mmol/L Iodoacetamide (IAM). Western blotting assay was performed with IgG2a mouse monoclonal antibody for glutathione‐protein complexes.

### 2.10 Determination of ROS production in UPEC BC1 treated with ebselen and silver ion

UPEC BC1 was grown until an OD_600 nm_ of 0.4 in LB medium and incubated with 80 μmol/L ebselen and 5 μmol/L silver ion for 20 minutes. The BC1 cells were stained with 10 μmol/L H_2_DCFH‐DA for 30 minutes at 37°C. After incubation, the ROS production was quantified by flow cytometry (BECKMAN COULTER, AW15093).

### 2.11 Acute mouse cystitis model assay

All experiments were performed in accordance with the relevant guidelines and regulations. Ninety healthy male BALB/c mice were randomly divided into six groups randomly (n = 15). The bladders of female mice were infected via transurethral catheterization by PE10 with 50 µL of MDR UPEC BC1 (×10^7^); and 20 mg/kg ceftazidime, 25 mg/kg ebselen, 6 mg/kg silver ion, 25 mg/kg ebselen plus 6 mg/kg silver ion, 10 mg/kg amikacin and DMSO were administered i.p. to separate groups on the 1st, 3rd and 5th days post‐infection. Seven days after the last treatment, the overall survival was calculated.

### 2.12 Mild mouse cystitis model assay

Sixty healthy male BALB/c mice were divided into six groups randomly (n = 10). The bladders of female mice were infected via transurethral catheterization with 50 µL of MDR UPEC BC1 (×10^6^). Group A was left infected as the control; groups B‐F were inoculated, and 20 mg/kg ceftazidime, 25 mg/kg ebselen, 6 mg/kg silver ion, 25 mg/kg ebselen plus 6 mg/kg silver ion, 10 mg/Kg amikacin and DMSO were administered i.p. on the 1st, 3rd and 5th days post‐infection, respectively. The bacterial loads were calculated by counting the colony formation unity (CFU) derived from homogenates of urinary tissue.

### 2.13 Immunohistochemical analysis

Mice bladders were bisected, fixed in formalin (10%), and embedded in paraffin. Paraffin sections (4 µm thick) were used to detect cytokine levels by immunohistochemical (IHC) analysis. Anti‐TNF‐α and anti‐IFN‐γ antibodies were used for the quantification of cytokines in tissue.

### 2.14 Routine urine and blood tests

Urine of mice was collected 2, 4 and 6 days post‐infection and diluted with normal saline to 1 mL, and the white blood cells (WBCs) and red blood cells (RBCs) in urine were determined by urine sediment. For investigation of liver and kidney function, blood was collected and centrifuged at 3000 rpm for 10 minutes. Serum alanine transaminase (ALT), aspartate aminotransferase (AST), urea and creatinine were determined.

### 2.15 Statistical analyses

Statistical analyses were performed by GraphPad Prism 6.0 (GraphPad Software). Means of data between two groups were contrasted using Student's *t* test. Sample rates between two groups were tested with chi‐square analysis. Overall survival was analysed by the Gehan‐Breslow‐Wilcoxon test. *P* values of <0.05 were considered to be significant.

## 3 RESULTS

### 3.1 Antibacterial activity of ebselen and silver ion against uropathogenic *E coli*

**FIGURE 1:**
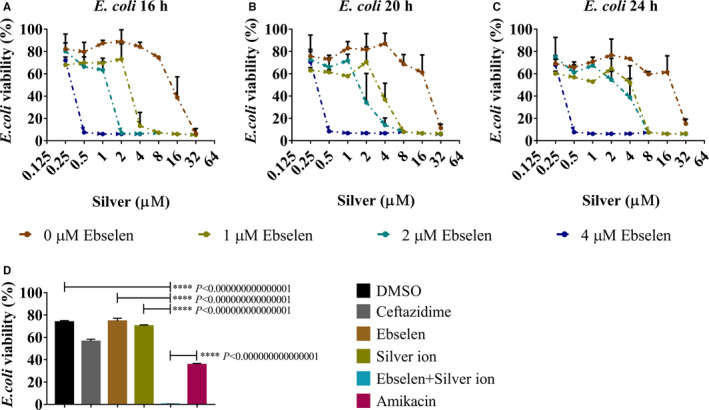
Antibacterial effect of ebselen and silver ion on UPEC BC1. UPEC BC1 overnight culture was diluted 1:100 and cultured with serial concentrations of ebselen and silver ion. A‐C, The OD_600 nm_ was measured at 16, 20 and 24 h post‐treatment; D, 64 μg/mL ceftazidime and 64 μg/mL amikacin were used as negative and positive controls, respectively, and a synergistic antibacterial effect of ebselen and silver ion was detected. (Student's *t* test. Data are presented as the means ± SD of three independent experiments)

The antibacterial effects of ebselen and silver ion on the growth of BC1 were detected by visible spectrophotometer. As shown in Figure 1A‐C, silver ion alone inhibited BC1 growth with a 90% minimal inhibition concentration (MIC_90_) of 32 μmol/L after incubation, while the addition of 4 μmol/L ebselen effectively reduced the MIC_90_ of silver ion to 0.5 μmol/L. Meanwhile, 4 μmol/L ebselen and 0.5 μmol/L silver ion showed no synergistic toxicity on human cells.^18^ These results demonstrate that ebselen and silver ion exerted significant selective synergistic toxicity against UPEC over mammalian cells. Furthermore, 64 μg/mL (109 μmol/L) amikacin was used as positive control, and 64 μg/mL (117 μmol/L) ceftazidime was used as negative control. The result in Figure 1D showed that 4 μmol/L ebselen and 0.5 μmol/L silver ion could completely inhibit bacterial growth and were more effective than amikacin (*P* < 0.00000000000001).

**FIGURE 2:**
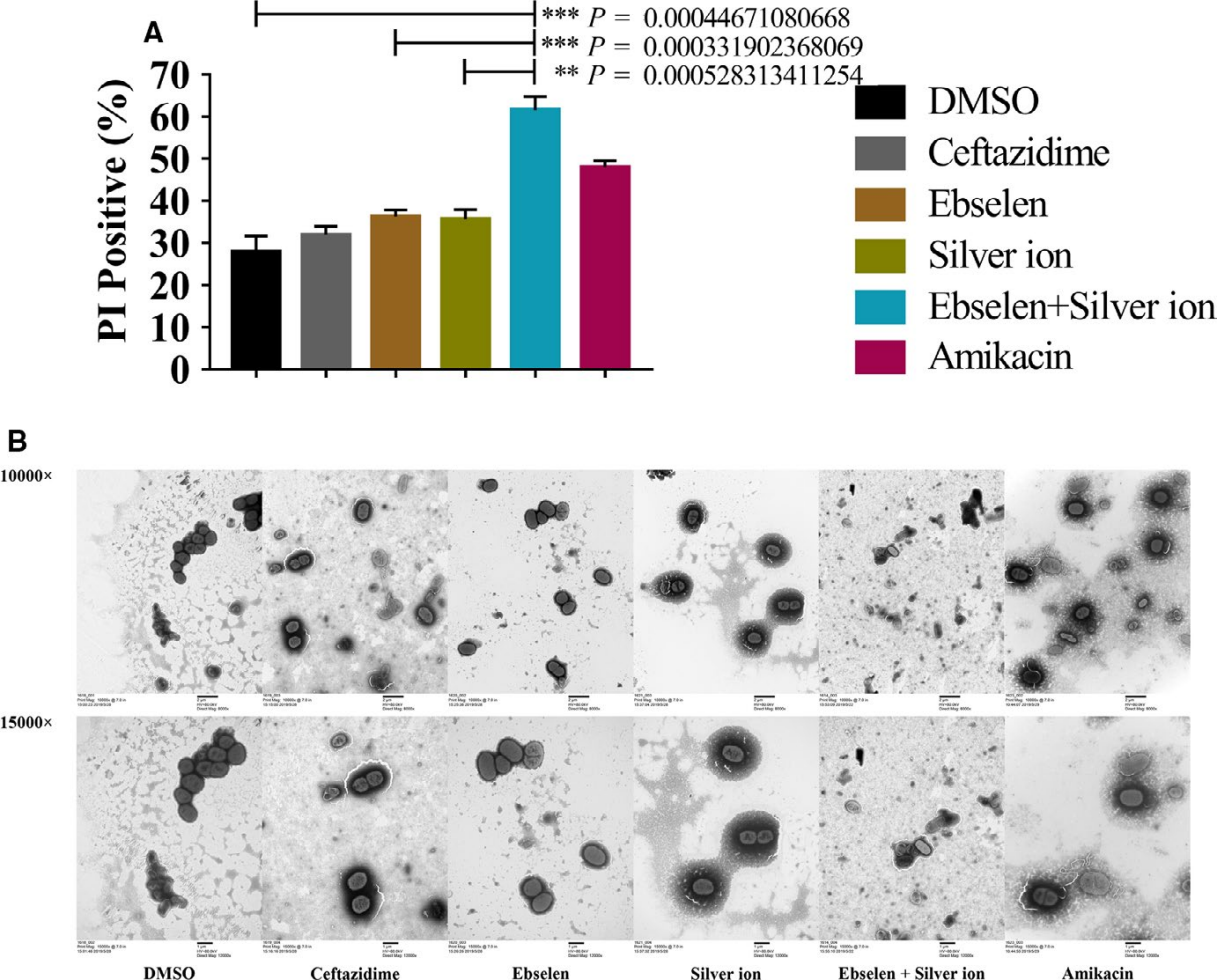
Antibacterial effect of ebselen and silver ion on UPEC BC1. UPEC BC1 was cultured until an OD_600 nm_ = 0.4 and treated for 20 min with DMSO, 640 μg/mL ceftazidime, 80 μmol/L ebselen, 5 μmol/L silver ion, 80 μmol/L ebselen plus 5 μmol/L silver ion or 640 μg/mL amikacin. Mean ± SD (A) (Student's *t* test. Data are presented as the means ± SD of three independent experiments.); B, Transmission electron microscopy of UPEC BC1 treated with ebselen and silver ion at 10 000× (upper row) or 15 000× (lower row) magnification

Propidium iodide (PI) nuclear staining, which reflects bacterial membrane permeability, was also performed. UPEC BC1 cells were grown until an OD_600 nm_ of 0.4 and further to be treated with 80 μmol/L ebselen and 5 μmol/L silver ion. In agreement with the inhibitory effect on bacterial growth, the percentage of PI‐positive cells exhibited the highest up‐regulation when BC1 cells were treated with 80 μmol/L ebselen and 5 μmol/L silver ion (65.73%), followed by 1.09 mmol/L amikacin (43.51%), 80 μmol/L ebselen (35.69%), 5 μmol/L silver (34.75%), 1.17 mmol/L ceftazidime (32.60%) and the control (17.43%) (Figure 2A; Figure S1). Thus, most cells were dead after ebselen and silver ion treatment, which showed significantly greater effect than did ebselen (*P* = 0.0003), silver ion (*P* = 0.0005) or the control (*P* = 0.0004). The results demonstrate that UPEC BC1 was highly sensitive to ebselen and silver ion in combination.

The effect of ebselen and silver ion on the morphology of UPEC BC1 was assessed by transmission electron microscopy (Figure 2B). The results showed that the morphology of UPEC BC1 changed significantly in the ebselen plus silver ion‐treated group compared with the control group. Normal BC1 has a smooth surface and a complete cell membrane and cell wall. After 20 minutes treatment with 80 μmol/L ebselen and 5 μmol/L silver ion, BC1 cells had obviously shrunk, cell membranes and cell walls were ruptured with a final cell death; meanwhile, 80 μmol/L ebselen, 5 μmol/L silver ion, 1.09 mmol/L amikacin and 1.17 mmol/L ceftazidime‐treated cells showed no obvious morphological changes.

### 3.2 Antibacterial activity of ebselen and silver ion targeting uropathogenic *E coli* TRX and GSH systems

**FIGURE 3:**
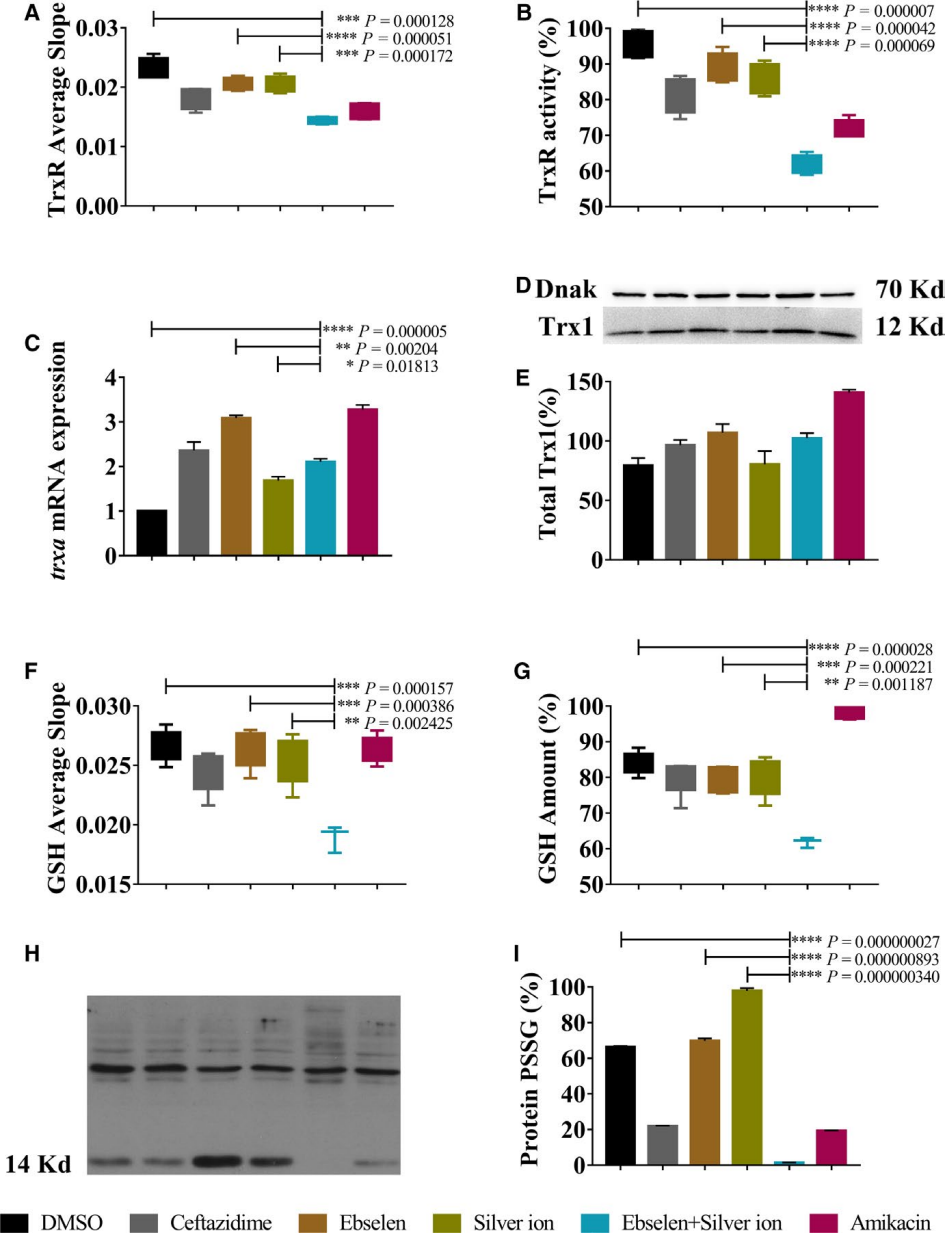
Antibacterial effect of ebselen and silver ion on UPEC BC1 targeting Trx and GSH systems. UPEC BC1 was cultured to OD_600 nm_ = 0.4 and treated with for 20 min with DMSO, 640 μg/mL ceftazidime, 80 μmol/L ebselen, 5 μmol/L silver ion, 80 μmol/L ebselen plus 5 μmol/L silver ion or 640 μg/mL amikacin. TrxR activity in slope (A) or end‐point (B) was detected using DTNB reduction assay in the presence of Trx in BC1 extracts; (C) *trxa* (Trx1) mRNA expression level was tested by qPCR (normalized levels with respect to the reference *rrs*); D and E, Trx1 protein level was measured by Western blot (normalized levels with respect to the reference DnaK); GSH amount in slope (F) or end‐point (G) was detected using DTNB reduction in the presence of GR in BC1 extracts; H and I, The *P*‐GSSG level of total proteins was detected by Western blot, and the expression level of protein in 14 kDa was qualified. (Student's *t* test. Data are presented as the means ± SD of three independent experiments)

The effects of ebselen and silver ion on bacterial TrxR activity, Trx expressions and total GSH level in UPEC BC1 were measured. The results showed that the combination of 80 μmol/L ebselen and 5 μmol/L silver ion efficiently inhibited the TrxR activity in UPEC BC1 when compared with the control (Figure 3A in slope*, P* = 0.0001) (Figure 3B in end‐point*, P* = 0.000007). Silver ion or ebselen alone also able to inhibit the TrxR activity, albeit less efficiently than the drugs in combination (Figure 3A in slope*, P* = 0.0001 or 0.00005) (Figure 3B in end‐point, *P* = 0.00004 or 0.00007). Meanwhile, although the qPCR assay showed that the relative expression level of *trxa* (encodes Trx1) was significantly different than for the control (*P* = 0.000005), ebselen (*P* = 0.002) or silver ion (*P* = 0.02) (Figure 3C), the protein level did not change after the treatment (Figure 3D,E). These results show that ebselen and silver ion affected Trx system bioactivity rather than protein expression.

Furthermore, 80 μmol/L ebselen plus 5 μmol/L silver ion were able to deplete GSH levels in UPEC BC1 significantly more than in the control (*P* = 0.0002 or 0.00003), silver (*P* = 0.002 or 0.001) or ebselen (*P* = 0.0004 or 0.0002) (Figure 3F in slope or Figure 3G in end‐point). Meanwhile, whether ebselen and silver ion depletion of GSH could affect protein *S*‐PSSG was also determined (Figure 3H,I). The result showed that protein *S*‐PSSG was reduced in bacteria treated with both ebselen and silver ion. The expression level of protein in 14 kDa was further qualified, and the results showed that bacteria treated with drugs in combination decreased the *S*‐PSSG when compared with silver ion (*P* = 0.0000003) or ebselen alone (*P* = 0.0000009), which further reflected the loss of GSH.

Taken together, the above results suggest that ebselen and silver ion inhibited Trx activity and depleted GSH in UPEC BC1.

### 3.3 The up‐regulation of the intracellular expression level of ROS

**FIGURE 4:**
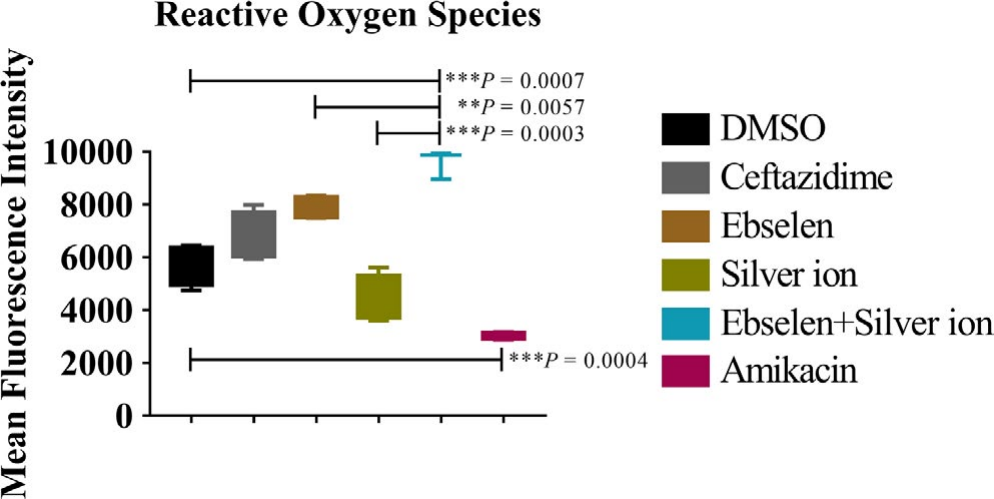
Antibacterial effect of ebselen and silver ion related to up‐regulation of ROS production level. UPEC BC1 was cultured to OD_600 nm_ = 0.4 and treated for 20 min with DMSO, 640 μg/mL ceftazidime, 80 μmol/L ebselen, 5 μmol/L silver ion, 80 μmol/L ebselen plus 5 μmol/L silver ion or 640 μg/mL amikacin. Mean fluorescent intensity (MFI) of means ± SD of H_2_DCF‐DA‐stained BC1 was detected to present ROS level. (Student's *t* test. Data are presented as the means ± SD of three independent experiments)

The mean fluorescent intensity (MFI) of ROS in BC1 cells was detected by flow cytometry, and the result showed that the ROS production level in BC1 cells treated with 80 μmol/L ebselen plus 5 μmol/L silver ion was clearly up‐regulated when compared with the control (*P* = 0.007), ebselen (*P* = 0.006) and silver ion (*P* = 0.0003) (Figure 4; Figure S2).

These results show that ebselen and silver ion constitute an effective antibacterial combination by inhibiting UPEC BC1 TrxR and depletion of GSH in vitro, and ROS production is one of the key mechanisms of its bactericidal activity.

### 3.4 The depletion of UPEC BC1 in the mouse cystitis model by ebselen and silver ion

**FIGURE 5:**
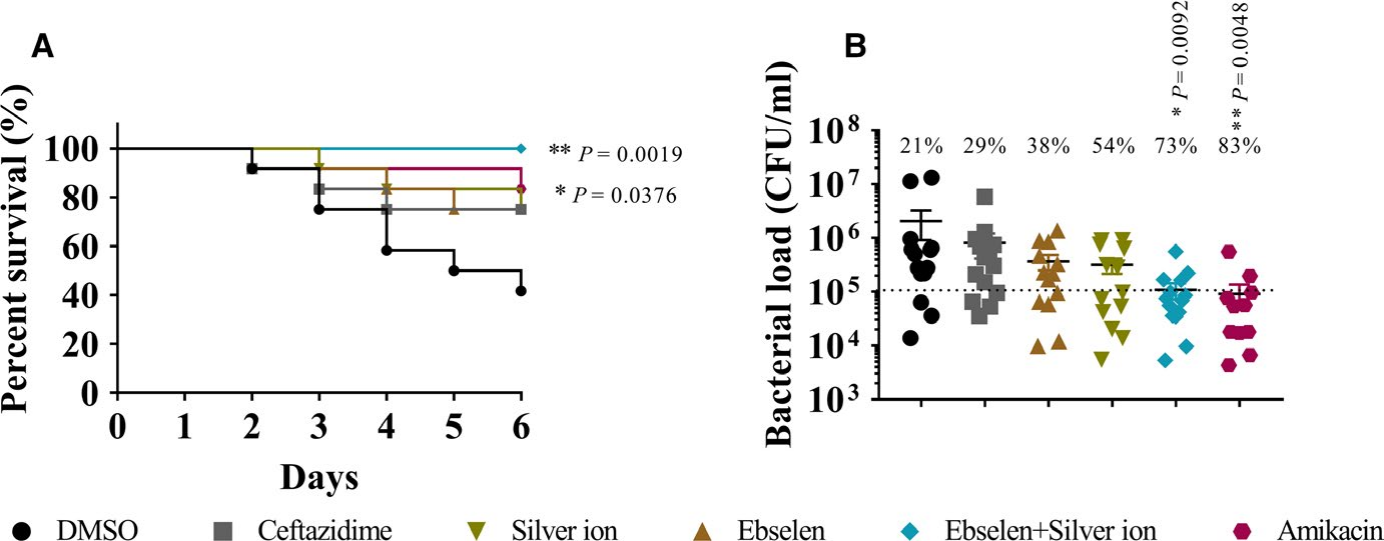
Therapeutic efficacy of ebselen and silver ion in treating UPEC BC1‐induced cystitis. A, MDR UPEC BC1 was cultured overnight, and the bladders of female mice were infected via transurethral catheterization with 50 μL 2 × 10^7^ CFU/50 μL to construct an acute cystitis model. Mice (n = 15) were injected ip with DMSO, 20 mg/kg ceftazidime, 25 mg/kg ebselen, 6 mg/kg silver ion, 25 mg/kg ebselen plus 6 mg/kg silver ion or 10 mg/kg amikacin on the 1st, 3rd and 5th days post‐infection. Overall survival was observed (data are presented by log‐rank (Mantel‐Cox) test). B, The mild cystitis model was established by transurethral catheterization with 50 μL 1 × 10^6^ CFU/50 μL. Mice (n = 10) were injected ip with DMSO, 20 mg/kg ceftazidime, 25 mg/kg ebselen, 6 mg/kg silver ion, 25 mg/kg ebselen plus 6 mg/kg silver ion or 10 mg/kg amikacin on the 1st, 3rd and 5th days post‐infection, and the bacterial load was calculated by counting the colonies derived from homogenates of urinary tissue (data are presented by chi‐square test)

Cystitis is among the most common bacterial infections of human. The mouse provides an excellent and tractable model system for cystitis caused by UPEC.^28^ As ebselen and silver ion have been demonstrated to be safe in our previous studies,^18^ mice were randomly divided into 6 groups, and 50 μL ~2 × 10^7^ CFU/50 μL or ~1 × 10^6^ CFU/50 μL BC1 were perfused via transurethral catheterization to construct acute and mild cystitis models, respectively. Mice were further injected i.p. with 25 mg/kg ebselen, 6 mg/kg silver ion, 25 mg/kg ebselen plus 6 mg/kg silver ion, 20 mg/kg ceftazidime, 10 mg/kg amikacin or DMSO. All mice with acute cystitis treated with silver ion plus ebselen survived, compared with 40% in the DMSO group (*P* = 0.0019) and 80% in the amikacin group (*P* = 0.038) (Figure 5A). Furthermore, the combination of ebselen and silver ion led to a significant reduction in bacterial load on‐site compared with the DMSO group (*P* = 0.092) (Figure 5B). Mice treated with ebselen or DMSO showed similar bacterial load after 72 hours of infection with UPEC BC1, whereas treatment with silver ion plus ebselen achieved a 73% reduction, followed by 54% in the silver ion group and 38% in the ebselen group (Figure 5B). Although amikacin showed an 83% reduction in bacterial load, it showed no significant difference with the drugs in combination (*P* = 0.005). These findings demonstrate the effective antibacterial effect of ebselen and silver ion against MDR UPEC BC1 in vivo.

### 3.5 The effects of ebselen and silver on inflammatory properties of mice with cystitis

**FIGURE 6:**
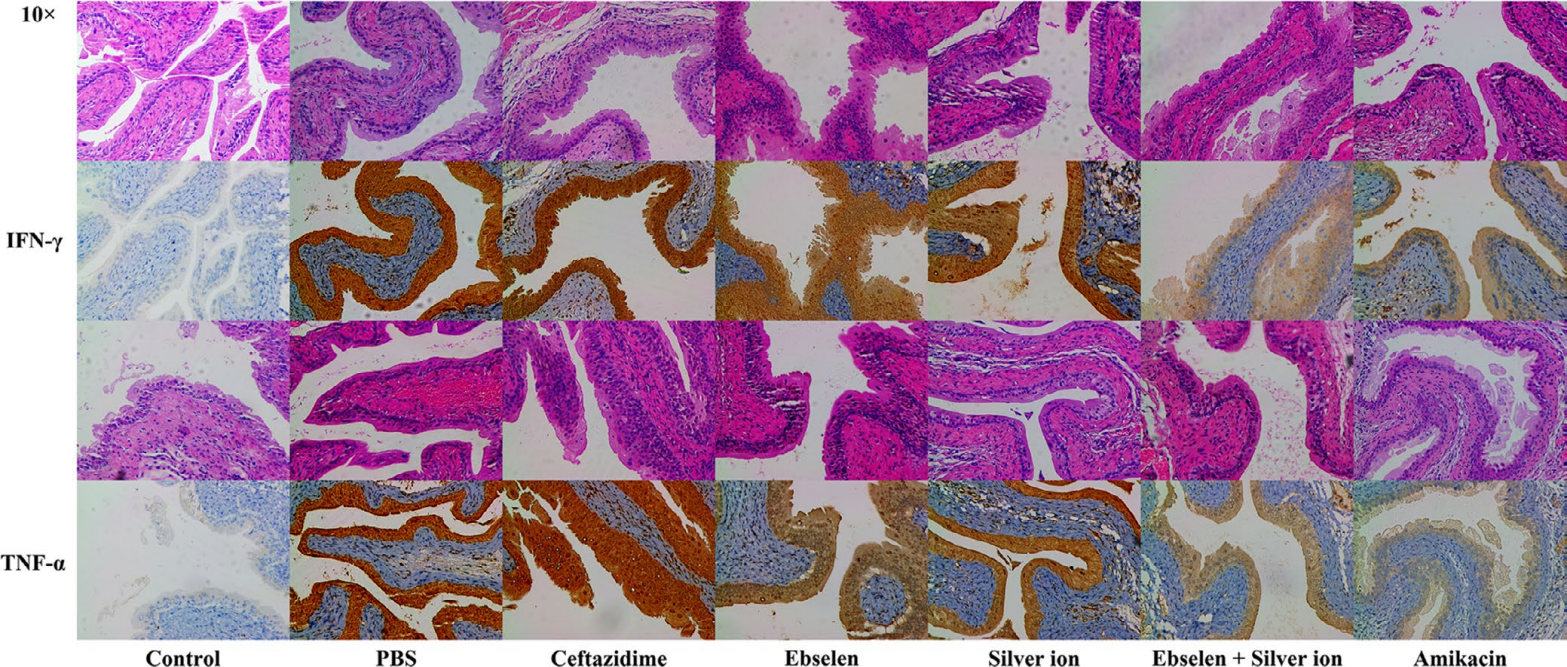
Immunohistochemical detection in mice treated with ebselen and silver ion following UPEC BC1‐induced cystitis mice. Bladders with different treatments (DMSO, 20 mg/kg ceftazidime, 25 mg/kg ebselen, 6 mg/kg silver ion, 25 mg/kg ebselen plus 6 mg/kg silver ion and 10 mg/kg amikacin) were used for pathological detection. The expression levels of IFN‐γ (upper rows) and TNF‐α (lower rows) are presented at 100× magnification

An IHC assay was used to measure the presence of pro‐inflammatory cytokines, including TNF‐α and FN‐γ. As shown in Figure 6, ebselen and silver ion significantly reduced the expression of both tested pro‐inflammatory cytokines compared to mice treated with DMSO. For TNF‐α, the group treated with ebselen plus silver ion exhibited the greatest reduction in expression, followed by amikacin and the control (Figure 6). For IFN‐γ, the group treated with ebselen plus silver ion also showed the greatest reduction in expression, followed by amikacin and DMSO (Figure 6).

**FIGURE 7:**
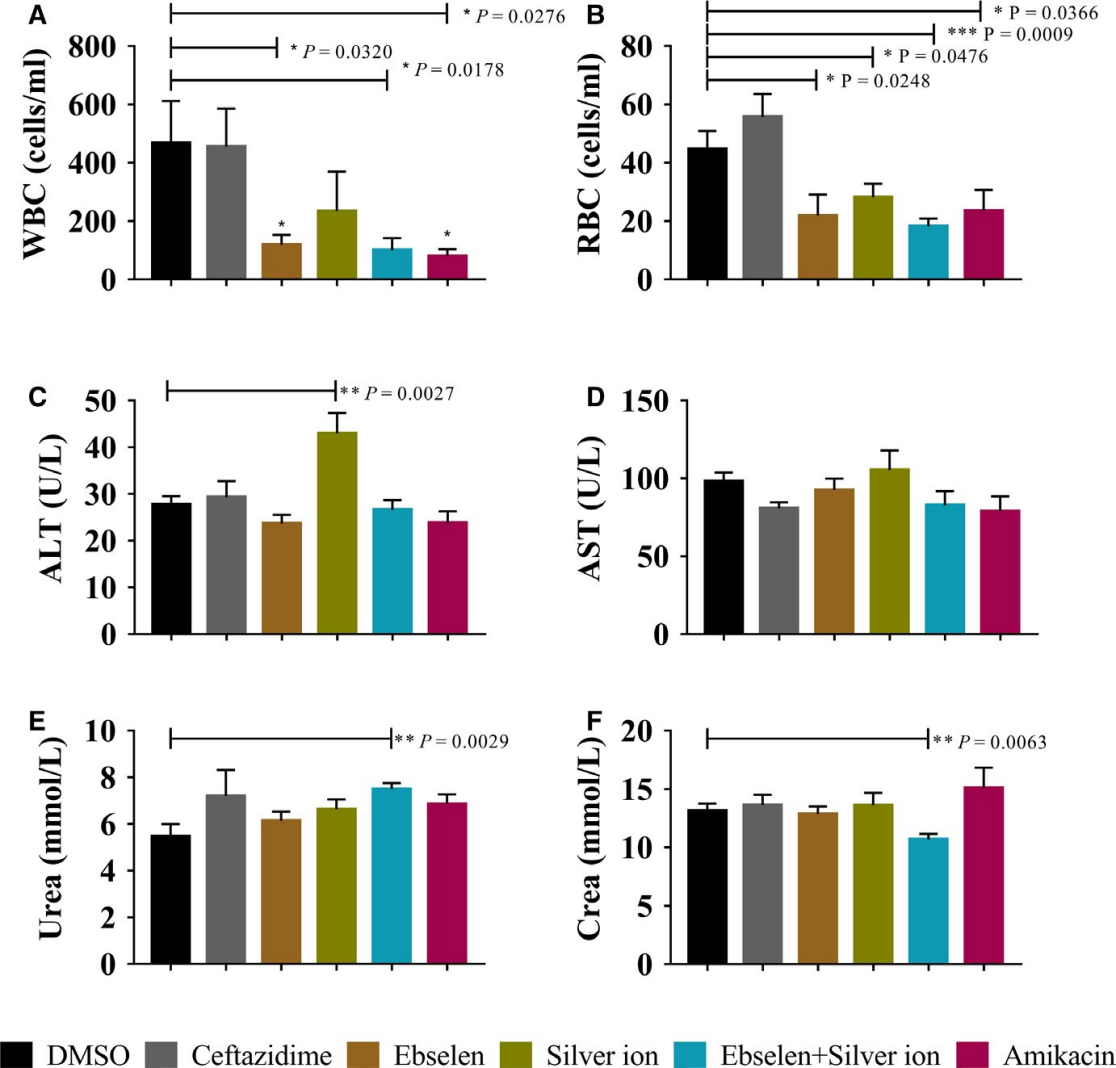
Routine urine and blood parameters for mice treated with ebselen and silver ion following UPEC BC1‐induced cystitis. Urine of mice was collected 2, 4 and 6 days post‐infection. The number of WBCs (A) and RBCs (B) of mice were determined by urine sediment. To evaluate the effects of drugs on the liver and kidney function of mice, the sera of mice were collected, and contents of ALT (C), AST (D), urea (E) and creatinine (F) were measured

The urine and peripheral blood from different groups of mice were collected. The WBCs and RBCs counts in urine were determined, and the results showed that ebselen and silver ion statistically reduced the numbers of WBCs (*P* = 0.03, Figure 7A) and RBCs (*P* = 0.0009, Figure 7B) compared with the control. Furthermore, ALT, AST, urea and creatinine in mouse blood serum were also detected, and the results showed that ebselen and silver ion treatment had no influence on the ALT (*P* > 0.05, Figure 7C) or AST (*P* > 0.05, Figure 7D) levels; meanwhile, the combined treatment up‐regulated the urea (*P* = 0.0003, Figure 7E) and down‐regulated the creatinine (*P* = 0.06, Figure 7F).

Overall, ebselen and silver ion treatment groups exhibited the greatest effects in reducing the UPEC BC1‐caused inflammation, and the activity of these drugs was considerably higher than that of amikacin. These results demonstrate that ebselen and silver ion may assist the recovery of infected urinary tracts, and their abilities to do so are considerably higher than that of amikacin.

## 4 DISCUSSION

Until now, UPEC has been the most common pathogenic bacteria,^29^ accounting for 95% of community‐acquired infections and 50% of hospital‐acquired infections.^30^ The failure of UTI treatment and the improper use of catheters are important risk factors for *E coli* bacteremia.^31^ UTI treatment has led to extensive use of antibiotics; the empirical antibacterial treatment of rUTI has especially led to a change in urinary bacterial ecology. Overuse of high‐efficiency broad‐spectrum antibiotics, such as fluoroquinolones, cephalosporin and aminoglycosides, has led to an increase in the frequency of MDR UPEC and has increased costs of treatment and hospitalization.^32^ In recent decades, the emergence and spread of MDR UPEC have developed into a huge clinical problem. In this study, the MDR UPEC BC1 strain was isolated from a clinical UTI patient. The strain was resistant to penicillin, cephalosporin, carbapenems, β‐lactamase inhibitors, aminoglycosides, quinolones, *etc* Its isolation demonstrated the universality of MDR UPEC in clinical settings, and it is urgent to develop new antibacterial strategies to fight against UPEC. In recent studies, we found out that ebselen and silver ion have potential antimicrobial activity against GSH‐positive bacteria, including MDR *E coli* and *A baumannii*.^18^, ^26^, ^27^ In this study, the bactericidal activity against MDR UPEC BC1 of ebselen and silver ion was confirmed by visible spectrophotometry, transmission electron microscopy, DTNB assay and flow cytometry in vitro. These results showed that ebselen and silver ion exerted potent bactericidal activity against the MDR UPEC BC1 strain, which was closely related to the bacterial TrxR inhibition and GSH depletion.

Since bacterial Trx and GSH systems are important for reducing various critical cellular antioxidants to maintain the intracellular redox environment balance, inhibition of TrxR and depletion of GSH are highly related to the excessive production of ROS.^18^ To detect the intracellular ROS expression, cells treated with ebselen and silver ion were stained by H_2_DCFH‐DA, and the FACS results confirmed that ebselen and silver ion inhibited electron transfer, which enormously influences the ROS removal. This result demonstrates that ROS elevation is a major player in determining the bacterial fates, which is consistent with many reports from other groups that ROS produced by some clinically used antibiotics contribute to their bactericidal efficacy.^24^, ^33^, ^34^

Using a well‐established model of experimental cystitis in which the bladders of female mice are infected via transurethral catheterization, the molecular details of the pathogenesis of bacterial cystitis have been substantially illuminated in the last decade. This promises to afford continued opportunity for discovery of pathogenic mechanisms and evaluation of therapeutic and preventive strategies for acute, chronic and rUTI. Here, the mouse models with acute and mild cystitis were developed with the UPEC BC1 strain, and the therapeutic efficacy of ebselen and silver ion on mouse total survival and bacterial burden were both measured. The results show that ebselen and silver ion possess significant therapeutic efficacy against MDR BC1‐caused cystitis, including the rescue of 80% of total mice from death, and can substantially (by 73%) reduce bacterial burden.

UPEC components, including lipopolysaccharides and pili, ligate Toll‐like receptors (primarily TLR4) on host epithelium and resident leucocytes,^35^ stimulating NF‐κB and other signalling pathways, eliciting the local secretion of inflammatory cytokines ^36^, ^37^ and drawing neutrophils to the infected tissues.^38^, ^39^, ^40^ UPEC adheres to bladder epithelial cells in a type I pili‐dependent manner and enters epithelial cells,^41^, ^42^ forming intracellular bacterial communities (IBCs).^43^ Their ability to attach and enter into cells in the bladder is a limiting step for their pathogenicity.^44^The immune response of the host can cause the bladder epithelial cells to fall off, so that IBCs are excreted with urine; meanwhile,^45^ the UPEC subsets exist as residual IBCs with resisting neutrophils and escape from IBS, and then invade the new bladder epithelial cells.^46^ Some IBCs members continue to infect the exposed immature bladder epithelium, and then form quiescent intracellular reservoirs (QIR) to escape immune clearance and resist systemic antibiotic therapy.^47^, ^48^, ^49^ These persistent UPECs will recur and cause recurrent cystitis. Under the influence of decreased immunity or exogenous inducements, these persistent UPEC will come back to life and cause recurrent cystitis.^37^, ^50^ In addition, UPEC can specifically up‐regulate indoleamine 2,3‐dioxygenase (IDO) within 1 hours after bladder invasion, resulting in the decrease of innate immune cell responses such as neutrophil recruitment to escape host immunity. It has been found that up‐regulation of IDO in a host induced by UPEC is related to the production of TNF‐α and IFN‐γ.^51^ Excessive inflammatory reaction during acute cystitis is related to more serious tissue damage, which facilitates the development of chronic infection by the host. After that, strong and sustained leucocyte reaction and inflammatory state may not help the host to clear the infection, but might inhibit the repair of exfoliated transitional epithelium, allowing UPEC to invade the deep epithelium.^52^, ^53^

In this experiment, we found that the pro‐inflammatory cytokines, WBCs and RBCs were significantly reduced following treatment with ebselen and silver ion. Declines in TNF‐α, IFN‐γ and WBCs in bladder may be highly related to the anti‐inflammatory effect of ebselen.^54^ Meanwhile, the decrease in RBCs in urine can be explained as follows: ebselen can inhibit endothelium‐mediated diastole by scavenging peroxides^55^ and inhibit oxidative stress of endothelial cells^56^ to alleviate the inflammation‐induced vasodilation‐related hematuresis in cystitis. Meanwhile, it can down‐regulate the levels of TNF‐α and IFN‐γ to reduce inflammatory injury. In addition, when ebselen and silver ion were used together, the ALT level showed no difference from that of the control group. This is because ebselen was able to alleviate the liver damage from different causes^57^, ^58^ and reduce the toxicity of heavy metals caused by methylmercury and manganese chloride in vivo, including silver ion.^22^, ^59^ The increase in blood urea has many causes, and it is not as useful a biomarker as serum creatinine for reflecting renal function. Thus, based on our results, ebselen and silver ion have no effect on liver and renal function. These results suggest that ebselen and silver ion may be able to serve as alternative agents to amikacin to help treat MDR UPEC BC1*‐*induced cystitis, and the sensitivity of bacteria to ebselen and silver ion is dependent on the antioxidant systems present in the bacteria.

## CONFLICT OF INTEREST

The authors declare that they have no conflict of interest.

## AUTHOR CONTRIBUTION

**Peng Wang:** Formal analysis (equal); Methodology (lead); Software (equal). **Jun Wang:** Project administration (lead); Writing‐review & editing (lead). **Zonglan Xie:** Data curation (lead); Software (supporting); Visualization (equal). **Jingxuan Zhou:** Investigation (lead); Visualization (equal). **Qianqian Lu:** Validation (lead). **Ying Zhao:** Software (supporting); Validation (supporting). **Chuanjiang Dong:** Resources (lead); Supervision (lead). **Li‐Li Zou:** Conceptualization (lead); Writing‐original draft (equal).

## Supporting information

Fig S1

Fig S2

## Data Availability

The data that support the findings of this study are available on request from the corresponding author. The data are not publicly available due to privacy or ethical restrictions.
