# Supplementary figures and images for: Depletion of multidrug‐resistant uropathogenic Escherichia coli BC1 by ebselen and silver ion
Source: J Cell Mol Med. 2020 Sep 25;24(22):13139–50. doi: 10.1111/jcmm.15920 (PMC7701569; doi:10.1111/jcmm.15920)

Control: P2

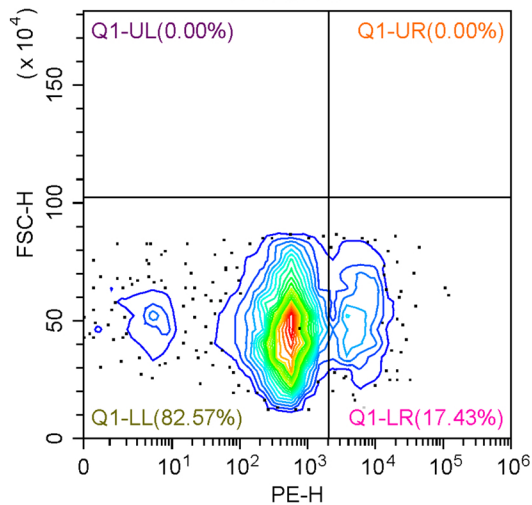

Ceftazidime: P2

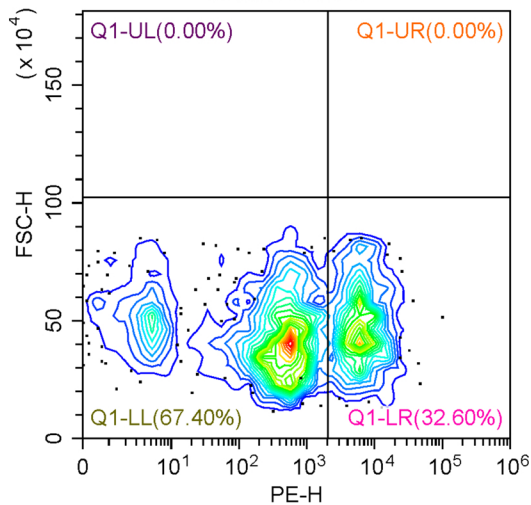

Ebselen: P2

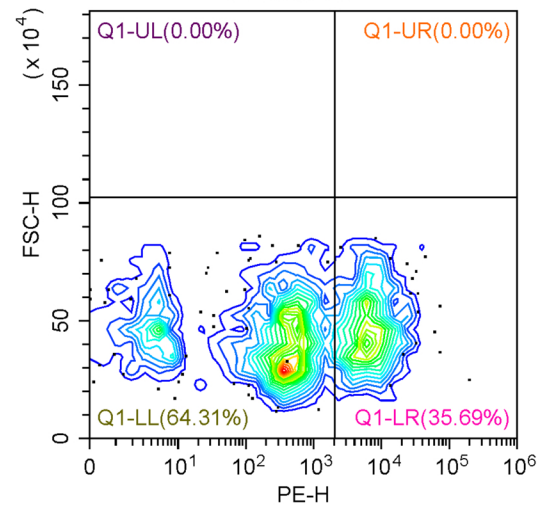

Silver ion: P2

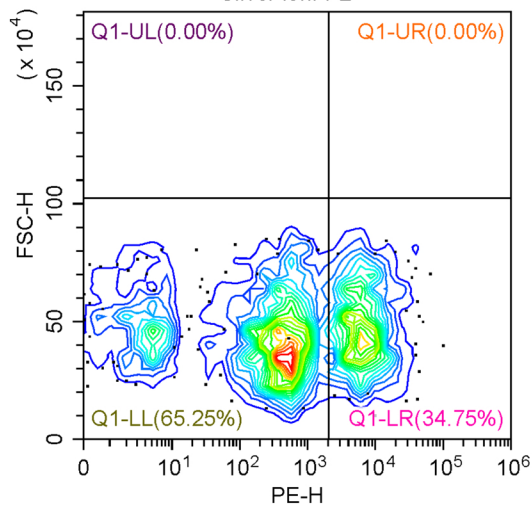

Ebselen + Silver ion: P2

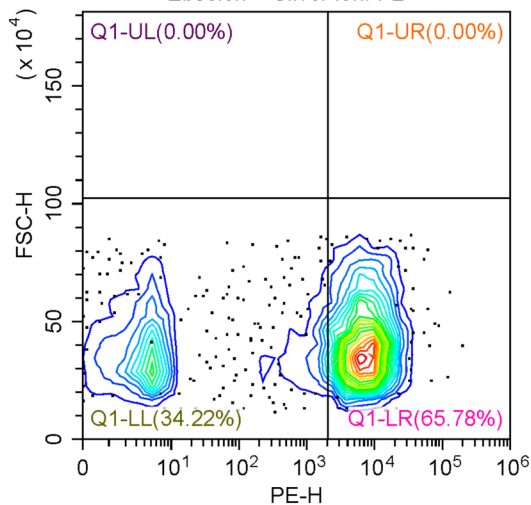

Amikacin: P2

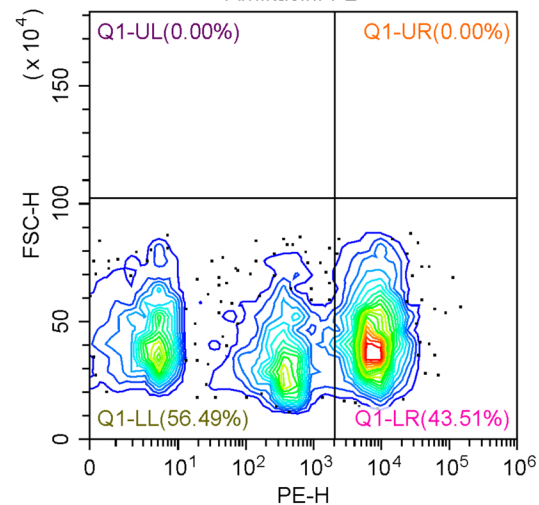

Supplement: Supplementary file 1 — Fig S1 [file JCMM-24-13139-s001.pdf]

Control: P2

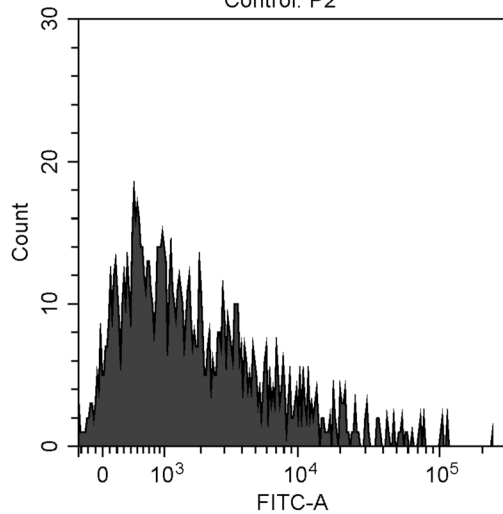

Ceftadime: P2

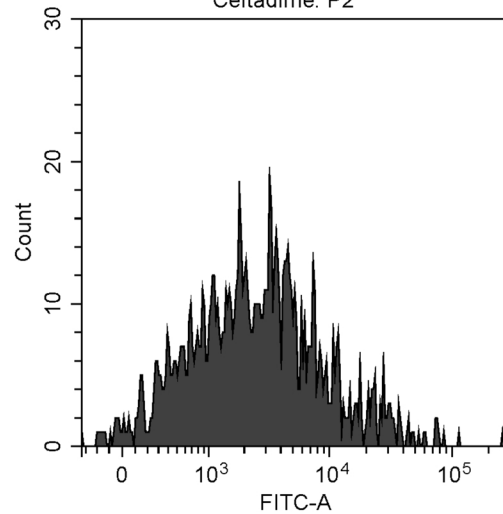

Ebselen: P2

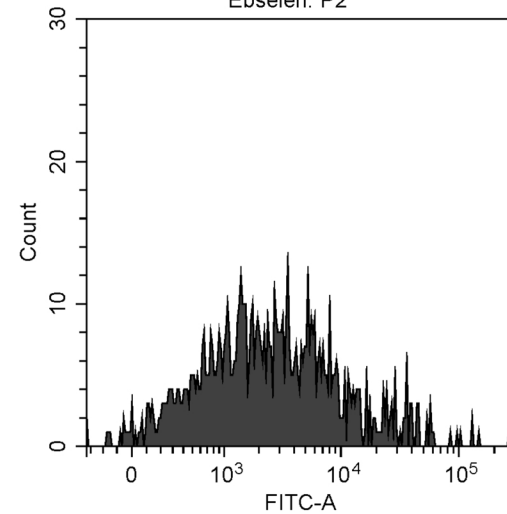

Silver ion: P2

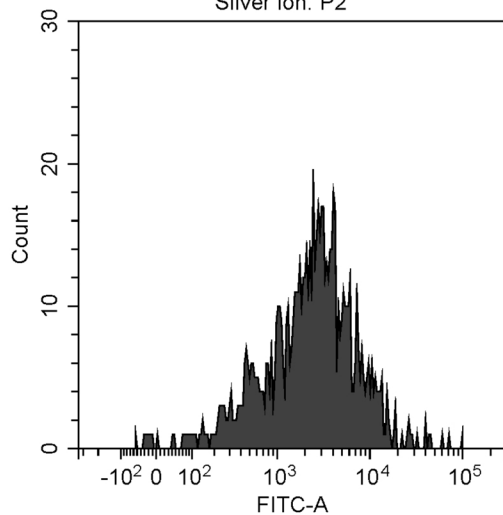

Ebselen + Silver ion: P2

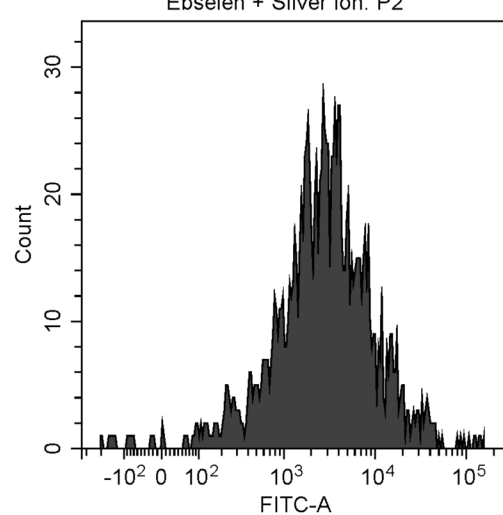

Amikacin: P2

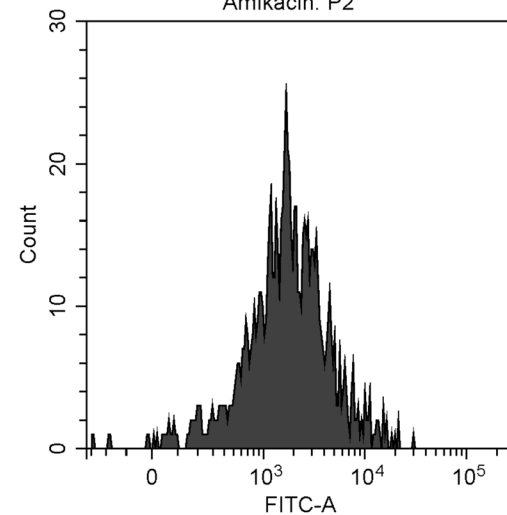

Supplement: Supplementary file 2 — Fig S2 [file JCMM-24-13139-s002.pdf]
